# The Moral of the Michigan Difficulty

**Published:** 1875-10

**Authors:** 


The Moral of the Michigan Difficulty.—The disturbance caused by the "inflation policy" of the legislature of Michigan in forcing a so-called homœopathic department into the regular school has reached the "correspondence" stage, so that we may hope the end is at hand. The only advantage to be hoped for from the letters is that the profession may be stirred up to take a firm position in the matter. Nothing is to be gained by temporizing: if the faculty does not appreciate that no compromise with quackery is possible, they will find out their error when it may be too late to repair it. If the medical department is thought worth preserving, a vigorous effort must be made. We appreciate that the position is a hard one. An ignorant legislature and public press have natural affinities with quackery, and the respectable man is always at a disadvantage in a dispute with an unscrupulous impostor; nevertheles the fight must be fought.

This affair, let it end as it may, is of great importance, as it points a moral that the profession at large should profit by. Some years ago a ring endeavored, happily without succes, to establish a national university under government control, and we occasionally hear suggestions of State boards to confer licenses to practice. Let it be understood once and for all that professional honor is too precious to be made one of the prizes of political contests. The theory as well as the practical working of our political system forbids us to consider government as a kind parent in whom we may trust; on the contrary, it can be kept pure only by the strongest and most persistent efforts, and a moment's inattention may give the intriguer years of advantage.

A legislature may be respectable one year, and the reverse the next; or if not the next, yet surely sooner or later; and it would be as futile as unbecoming for us to pit ourselves against the quack in lobbying and bribery. Professional affairs are safe only in our own hands; let us keep them there.—*Boston Med. and Surg. Jour.*

